# Demographic, Geographic, and Temporal Patterns of Adult Dental Emergency Presentations in Arad County, Western Romania

**DOI:** 10.3390/healthcare14101366

**Published:** 2026-05-15

**Authors:** Mihaela Cristina Negru, Andreea Mihaela Banta, Mirela Voicu, Dragoş Vasile Nica, Ioana-Cristina Talpoș-Niculescu, Iustin Olariu

**Affiliations:** 1 Department of ENT, “Victor Babeş” University of Medicine and Pharmacy, Eftimie Murgu Square No. 2, 300041 Timișoara, Romania; mihaelaprodea@umft.ro (M.C.N.); andreea.banta@umft.ro (A.M.B.); 2 Faculty of Pharmacy, “Victor Babeş” University of Medicine and Pharmacy, Eftimie Murgu Square No. 2, 300041 Timișoara, Romania; 3 Research Center for Pharmaco-Toxicological Evaluations, Faculty of Pharmacy, “Victor Babeş” University of Medicine and Pharmacy, Eftimie Murgu Square No. 2, 300041 Timișoara, Romania; nicadragos@gmail.com; 4 Research Center in Dental Medicine Using Conventional and Alternative Technologies, Department of Prostheses Technology and Dental Materials, Faculty of Dental Medicine, “Victor Babeş” University of Medicine and Pharmacy, 9 Revoluției 1989 Blvd., 300070 Timișoara, Romania; 5 The National Institute of Research—Development for Machines and Installations Designed for Agriculture and Food Industry (INMA), Bulevardul Ion Ionescu de la Brad 6, 077190 București, Romania; 6 Discipline of Orodental Diagnosis and Ergonomics, Faculty of Dental Medicine, “Victor Babeș” University of Medicine and Pharmacy, 9 Revoluției 1989 Blvd., 300070 Timișoara, Romania; 7 Department of Dentistry, Faculty of Dentistry, “Vasile Goldiş” Western University of Arad, 94–96 Revoluţiei Blvd., 310025 Arad, Romania; olariu.iustin@uvvg.ro

**Keywords:** dental care, pulpitis, dental caries, emergency service, periapical periodontitis, periapical abscess, toothache, rural health

## Abstract

**Background/Objectives**: This retrospective study aimed to assess the sociodemographic and temporal factors associated with dental emergency presentations in adults from Arad county, Western Romania. **Methods**: We collected data on age, sex, origin area, presentation time, and diagnostic (macro)category in 510 adult patients. Diagnoses were grouped into four macrocategories: dental pathology (PD), endodontic and periapical pathology (EPP), odontogenic septic complications (OSC), and other emergencies (OE). **Results**: EPP was the predominant (macro)category, with most cases involving acute apical periodontitis and pulpitis. Age differed significantly across diagnostic categories (*p* < 0.001), with PD patients being significantly younger than other groups (*p* < 0.001). Increasing age was associated with higher odds of EPP (AOR = 1.06, *p* = 0.004) but lower odds of PD (AOR = 0.93, *p* < 0.001). Rural origin was associated with increased odds of EPP (AOR = 1.49, *p* = 0.031) but decreased odds of OE (AOR = 0.39, *p* = 0.003). No significant associations were identified for sex. Most patients presented in the evening (46.47%) and on weekends, particularly Sundays (21%; *n* = 107) and Saturdays (16%; *n* = 82). Patient age differed significantly across time intervals (*p* = 0.016), with individuals seeking dental emergency care during morning and afternoon hours being significantly older than those presenting in the evening (*p* = 0.009) or nighttime (*p* = 0.047). **Conclusions**: Our findings suggest that observed sociodemographic and temporal differences may be associated with variations in the stage and type of dental pathology at presentation. Understanding these presentation patterns may help inform service organization and resource allocation in emergency dental care.

## 1. Introduction

Dental emergencies represent a major component within the spectrum of dental conditions—the most widespread non-communicable diseases [1,2]. These presentations exert a substantial financial burden on national budgets and often represent the “tip of the iceberg” in a system where prevention has failed or is unaffordable for economic reasons [3,4,5]. Taken together with an aging population and increasing treatment complexity [6], these circumstances have made emergency dentistry an important indicator of systemic efficiency and social equity in the contemporary public health landscape in Europe, not just a simple technical branch of dentistry [7,8].

Romania exhibits one of the highest levels of unmet dental care needs in the European Union, as well as the greatest socioeconomic disparity on this topic [9]. This negative outcome is likely related to the systemic challenges and transformations encountered over the past quarter of a century by the oral health landscape in Romania, especially the predominance of the private sector, chronic underfunding, limited reimbursed procedures, and the low number of dentists contracting with the public health insurance system [10]. However, existing research is fragmented and regionally concentrated. Most studies are local (single-center), primarily retrospective descriptive and limited to local emergency units [5,7,11,12,13,14,15,16,17,18]. National epidemiological data are also scarce [10], with no studies on nationwide emergency dentistry registry or multicenter analyses [9]. In addition, there is little information about socioeconomic determinants of emergencies (e.g., income, education, geographic location) or predictors of emergency presentation (e.g., age, sex, smoking, comorbidities) [14,16]. Moreover, information on when patients arrive (hours/day) is poorly documented, although it is essential for resource planning [19].

This study aimed to identify the prevalence and distribution of dental emergencies and their relationship to key demographic factors among patients seeking unscheduled urgent dental care at the Arad County Emergency Clinical Hospital (Arad county, Romania). This county may serve as an informative regional model due to its mixed urban–rural population, strategic trans-European geographic position, and reliance on a national dental system dominated by the private sector [10,20,21]. These features make it suitable for examining how emergency dental services function under constrained public-sector availability. The need for the study in this area is accentuated by recent regional data indicating major gaps in prevention: in Western Romania, the prevalence of active caries reaches alarming levels of 71.2% among schoolchildren, potentially reflecting limitations in current preventive oral health strategies and increased pressure on emergency services [10]. In this context, we aimed to address this lack of multicenter, nationwide data in Romania and the scarcity of robust information on the sociodemographic and temporal determinants of dental emergency presentations in the region. To achieve this, we provide a structured analysis of demographic, residence-based, diagnostic, and temporal presentation patterns in a major 24 h emergency dental center from Arad County, thereby offering clinically relevant insights for service organization and future regional comparative research.

## 2. Materials and Methods

### 2.1. Study Design

This was a retrospective cross-sectional observational study conducted at the Arad County Emergency Clinical Hospital (Arad county, Romania)—a large hospital in the western part of Romania that covers a population exceeding 400,000 inhabitants. It functions as the only 24 h dental emergency center in the Arad county, backed by a team of five certified dentists [22]. The study received approval from the Institutional Ethics Committee at the aforementioned hospital (approval No. 123/2 March 2026) and was conducted in agreement with the Declaration of Helsinki (2008), including subsequent amendments [23].

### 2.2. Participants and Eligibility Criteria

We retrieved information on patients presenting for emergency dental and oral care between 17 September and 23 December 2025. Collected medical data included sex, age, origin area, time of presentation at the emergency department (hour and day), and diagnosis. The rationale behind using only sex, age, and area of residence stems from their routine recording in clinical settings and well-established effect on healthcare utilization patterns and access to dental care.

The primary inclusion criteria were aged 18 years or older, availability of clinical and demographic data, and first eligible presentation. The unit of analysis was the emergency dental presentation, not the individual patient. To reduce duplicate counting, when the same patient presented more than once on the same day for the same clinical condition, only the first presentation was retained. Presentations occurring on different days or for different diagnoses were considered separate emergency episodes.

Patients younger than 18 years, with incomplete records, non-dental complaints, or insufficient data for diagnostic classification were excluded. Furthermore, we excluded individuals with diagnoses that did not constitute medical emergencies, including unfinished fillings, dental plaque and calculus, oral thrush, chronic gingivitis, and orthodontic discomfort without complications. Non-emergency uncomplicated eruption complaints were also excluded, while complicated/urgent eruption-related cases were retained.

Individual diagnoses were grouped into four (macro)categories based on etiopathogenic and therapeutic criteria and aligned with ICD-10 codes: (i) dental pathology (PD), including deep caries; (ii) endodontic and periapical pathology (EPP), including all types of pulpitis and apical periodontitis; (iii) odontogenic infections and septic complications (OSC), including all types of abscesses and cellulitis; and (iv) other emergencies (OE), including traumatic dental injuries, temporomandibular disorders, pericoronitis, alveolitis, hemorrhagic complications, and insufficiently specified urgent dental conditions. Trauma-related diagnoses were harmonized under ICD-10 S02.x codes when documentation indicated acute traumatic origin. ICD-10 coding was applied pragmatically to support retrospective epidemiological categorization within emergency clinical documentation, prioritizing consistency and functional grouping over subspecialty-level diagnostic granularity.

### 2.3. Statistical Analysis

We first used a Kruskal–Wallis test to assess intergroup differences in age. Dunn’s tests with Bonferroni correction were applied for pairwise comparisons in case of significant results. Differences in the distribution of sex (male vs. female) and origin area (urban vs. rural) were determined using Chi-square tests based on 4 × 2 contingency tables [24]. For significant overall Chi-square associations, post hoc pairwise comparisons between diagnostic macrocategories were performed using separate 2 × 2 Chi-square tests. To control for inflated Type I error due to multiple testing, Bonferroni correction was applied by adjusting the statistical significance threshold according to the number of pairwise comparisons performed (0.05/6 comparisons, i.e., *p* < 0.0083). This conservative correction strategy was selected to ensure methodological transparency, maintain consistency with other post hoc analyses performed in the study, and minimize the likelihood of false-positive findings. Non-significant comparisons were interpreted cautiously, and no borderline or trend-level associations were considered statistically meaningful after correction.

We then conducted separate binary logistic regressions to evaluate the independent associations between age, sex, and area of residence with each dental (macro)category. Age was modeled as a continuous predictor, while the female sex and urban residence served as reference categories. Multicollinearity was assessed using variance inflation factors (VIF), whereas model calibration was evaluated using the Hosmer–Lemeshow goodness-of-fit test. The aforementioned strategy (separate binary logistic regression models for each stratum) was selected to maximize category-specific interpretability, preserve statistical stability across uneven subgroup sizes, and allow clinically meaningful evaluation of independent predictors for each emergency macrocategory. Because the four diagnostic (macro)categories represented distinct clinical entities with differing pathophysiological progression, independent binary models allowed each stratum to be directly compared against all remaining presentations. This facilitated clearer identification of predictors uniquely associated with each form of emergency dental pathology. This approach also enhanced clinical interpretability by providing straightforward adjusted odds ratios for each macrocategory individually, rather than relying on indirect intercategory comparisons inherent to multinomial models. Moreover, given the substantial imbalance in subgroup frequencies, separate binary models improved statistical robustness by reducing potential instability, coefficient distortion, or reduced precision that may arise when applying multinomial logistic regression to unevenly distributed outcomes.

Patients were stratified based on the time interval when they sought emergency dental care into three strata: (i) morning/afternoon (08:00–16:00); (ii) evening (16:00–00:00); and (iii) night (00:00–08:00). Kernel density estimation (KDE) smoothing was applied for descriptive visualization of hourly presentation patterns. Differences in age across time intervals were assessed using the Kruskal–Wallis test, followed by Dunn’s post hoc tests with Bonferroni correction in case of significant differences. A *p*-value less than 0.05 was considered significant [25], except for post hoc analyses conducted for categorical variables (see above). All statistical analyses were performed using Stata v11 (StataCorp, College Station, TX, USA).

## 3. Results

The distribution of specific conditions within each (macro)category is presented in Table 1. EPP represented the predominant (macro)category, with most cases involving acute apical periodontitis, followed by pulpitis, and acute exacerbations of established chronic apical periodontitis lesions. In contrast, the non-exacerbated chronic form was rare. We also found a substantial prevalence of OSC, primarily abscesses and cellulitis, but a reduced prevalence of PD.

The distribution of cases across the four dental (macro)categories (types of dental emergency) is shown in Table 2. We observed significant age differences (Kruskal–Wallis test, *p* < 0.001), with patients with PD being significantly younger than those from the other (macro)categories (Dunn’s test with Bonferroni correction; in all cases *p* < 0.001). The other paired comparisons yielded no significant differences (Dunn’s test with Bonferroni correction, *p* ≥ 0.144).

Origin area was significantly related to the distribution of cases across the four dental (macro)categories (Chi-square test, *p* = 0.011), whereas sex did not (Chi-square test, *p* = 0.568). Post hoc examination of urban–rural differences revealed only one significant comparison; that is, the comparison between PD individuals and OE individuals (Chi-square test with Bonferroni correction, *p* = 0.003). Comparisons between OE and both EPP (Chi-square test with Bonferroni correction, *p* = 0.015) and OSC (Chi-square test with Bonferroni correction, *p* = 0.073) yielded relatively lower corrected *p*-values, suggesting potentially meaningful distributional differences that did not reach formal statistical significance after adjustment. All remaining pairwise comparisons yielded substantially higher corrected *p*-values (Chi-square tests with Bonferroni correction, *p* ≥ 0.274).

Regression model diagnostics supported the adequacy and stability of all analyses. Multicollinearity assessment revealed negligible predictor overlap, with variance inflation factor values close to 1 for all variables (age = 1.002, sex = 1.001, rural residence = 1.002). Event-per-variable ratios exceeded the conventional minimum threshold of 10 events per predictor across all models, confirming sufficient statistical stability despite subgroup size variability: PD, 14.3 (43 cases); EPP, 99.0 (297 cases); OSC, 36.3 (109 cases); and OE, 20.3 (61 cases). Model calibration was acceptable for all logistic regression models, with non-significant results throughout (Hosmer–Lemeshow goodness-of-fit test: PD, *p* = 0.395; EPP, *p* = 0.259; OSC, *p* = 0.427; and OE, *p* = 0.449). (macro)category are given in Table 3. Age was a significant predictor of EPP, with each one-year increase being linked to a 5% increase in the odds of this outcome. Origin area exerted a similar effect; that is, individuals from rural areas had nearly one and a half times the likelihood of having these pathologies. On the other hand, increasing age was independently associated with significantly lower odds of having PD. Moreover, rural origin was associated with a significantly reduced likelihood of falling into the OE (macro)category. No independent associations were identified for the other variables.

**Figure 1:**
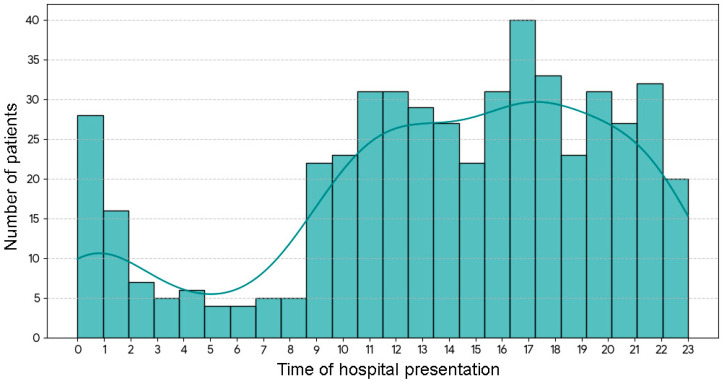
Temporal distribution of dental emergency presentations. Bars represent hourly patient frequencies, while the superimposed KDE curve illustrates the smoothed temporal distribution pattern.

The temporal dynamics of presentations to dental emergency services is illustrated in Figure 1. Most patients sought emergency dental care in the evening (46.47%, *n* = 237), followed by morning/afternoon (37.25%, *n* = 190), whereas the smallest proportion of cases was recorded at night (16.27%, *n* = 83). The analysis of hourly patient arrival patterns demonstrated substantial variation across the full 24 h day, with a peak in the evening (16:00–24:00) when 46.47% of patients presented for dental emergency care.

Patient age differed significantly across time intervals (Kruskal–Wallis test, *p* = 0.016). Individuals seeking dental emergency care in the morning/afternoon were significantly older [median age: 33 (22–50)] than those accessing these services in the evening [median age: 27 (18–37)] or at night [median age: 27 (19–41)]. We observed significant differences when comparing the first group with the second group (Dunn’s test with Bonferroni correction, *p* = 0.009) and the third group (Dunn’s test with Bonferroni correction, *p* = 0.047). However, no significant differences were observed between the age of patients presenting to dental emergency in the evening and at night (Dunn’s test with Bonferroni correction, *p* = 0.561). With respect to weekly distribution of dental emergency presentations, the highest number of presentations occurred on Sundays (*n* = 107, 21.00%), followed by Saturday (*n* = 82, 16.00%), Monday (*n* = 77, 15.00%), Thursday (*n* = 71, 14.00%), Friday (*n* = 77, 15.00%), with the lowest attendance observed on Wednesdays (*n* = 56, 11.00%) and Tuesdays (*n* = 40, 8.00%).

## 4. Discussion

Derived from an underrepresented region in Romania, this study expands our understanding of emergency dental care in Romania specifically and Eastern Europe broadly. The present findings may be indicative of limited access to preventive care and increasing reliance on emergency services—features typical for post-transition healthcare systems [10,15]. Notably, this investigation is built on a pragmatic classification into macrocategories aligned with ICD-10, reducing diagnostic fragmentation and allowing multivariate (logistic) modeling of predictors (age, sex, residency). In fact, the variability of definitions/codes and reporting methods is considered a major problem that hinders comparisons between centers and over time [26]. Our strategy may therefore be of notable translational and clinical importance since most existing studies in Romania report nominal diagnoses (e.g., pulpitis, apical periodontitis, abscess) without a comparable aggregation scheme between studies [11,12,13,14,15,16,17,18].

Our results reveal that acute inflammatory processes of dental pulp and periapical tissues are the main reasons for presentation to dental emergency services. On the other hand, the relatively high proportion of odontogenic infections indicates that untreated lesions tend to progress to suppurative complications within the analyzed cohort. In contrast, we observed a lower contribution of uncomplicated carious pathology and other dental emergencies. This pattern is broadly consistent with the trends reported in dental literature. For example, a large retrospective study from a public hospital dental emergency service in Târgu Mureș (2012–2017) reported 38,610 presentations, with a distribution of diagnoses dominated by “pulp infections” (30.33%) and “periodontal infections” (23.08%), followed by dental abscesses (12.75%) and root remnants (13.29%) [16]. A post-COVID Romanian study (Bihor/Oradea, 2022–2023) analyzed 4769 patients and showed that the most frequent diagnoses were acute pulpitis (39.2%) and acute apical periodontitis (37.5%); hence, over three-quarters of cases in the endodontic/periapical sphere [14]. Moreover, a population-based study (2010–2022) conducted in the Czech Republic and based on a registry of reimbursed health services reported again the predominance of endodontic and periapical outbreaks [27]. Overall, these data may reflect delayed presentation, with patients postponing care until caries progresses to symptomatic pulpitis or apical periodontitis.

Consistent with literature data [28,29,30,31], patients with dental pathology formed a distinct and significantly younger demographic group than the other conditions. The natural history of dental disease accounts, at least partly, for this disparity in age groups. Thus, dental pathologies (including cavities) are the entry point, while endodontic and septic pathologies are the end-stage complications. The time interval required for bacteria to dissolve enamel and penetrate dentin is typically several years, and as a consequence, the observed age difference aligns with the established clinical timeline of caries progression to pulpal involvement [32]. Existing literature shows that the third decade of life (20–29 years) corresponds to the peak period for primary caries and secondary caries (decay around existing fillings) [33]—a finding in line with our data. If those early lesions are not treated or restorations fail, the infection progresses from a localized process, confined to the hard tissues of the tooth, to dental pulp and periapical structures, triggering pulpitis or apical periodontitis (endodontic pathologies) [34,35]. Because this “penetration” takes time, the average age for these complications naturally pushes towards the fourth decade of life (30–39 years). This decade is also the point when most patients typically receive their first major restorations (fillings). With common fillings lasting between 5 and 15 years, many fillings fail or leak in the fourth decade of life [36]. At this stage, the decay is often deeper, closer to the nerve, and likely to require endodontic intervention or result in a septic (abscessed) state [37]. These differences may also stem from age-related changes in pulp morphology [38,39]; these changes can hide the pain of a slow-growing cavity, allowing chronic infection and sepsis to develop before symptoms appear.

Notably, a minority of patients presented with other emergencies. These conditions, however, may yield a disproportionate burden of long-term disability and medico-legal complexity since this (macro)category includes traumatic dental injuries, temporomandibular disorders, and other potentially disabling conditions. In fact, evidence from an adolescent Portuguese cohort revealed that specific injury types (e.g., tooth and alveolar loss, complicated fractures, temporomandibular injuries) predict the duration of temporary disability and the risk of definitive damage [40]. Moreover, longitudinal studies on temporomandibular trauma indicate that acute injuries initially managed in emergency settings may evolve into chronic dysfunction requiring specialized rehabilitation [41].

Age was associated with increased odds of endodontic and periapical pathology and lower likelihood of having dental pathology. This pattern may reflect the natural history of dental disease progression; that is, early-stage lesions (confined to enamel and dentin) are more common in younger individuals, whereas older patients are more likely to present with the sequelae of long-standing, untreated disease [38,39]. In addition, cumulative exposure to risk factors and potential delays in accessing dental care may contribute to the higher burden of advanced pathology in older populations [42].

We identified clinically meaningful differences along the urban–rural axis, supporting that the distribution of dental cases across geography may reflect a genuine association rather than chance. The only significant difference was observed between dental pathology and other emergencies: urban inhabitants showed a higher prevalence of the latter (macro)category—a heterogeneous category that reflect variations in the perception of urgency, accessibility, and access to primary dental care. This emphasizes the need to better characterize these cases and optimize patient flow, especially for populations from different backgrounds.

Interestingly, rural origin was linked to increased odds of having endodontic and periapical pathology, but reduced likelihood of presenting with conditions classified as other emergencies. It is possible that, depending on the area of origin, the simpler dental issues might be handled differently or reported at different rates [5]. With more dentists per capita, appointments may be more easily accessible in urban centers, whereas travel time is negligible. This may encourage patients to seek help for minor dental complaints (e.g., tooth sensitivity or minor restorative irregularities) rather than more severe emergencies (e.g., spreading odontogenic infections or traumatic dental injuries) [43], increasing their likelihood to be diagnosed with enamel- or dentin-level lesions. These non-pulpal dental lesions typically involve simple restorations—like a filling or a sealant—while preserving tooth vitality [44]. In contrast, rural patients tend to present for dental care with severe tooth pain and endodontic pathology (pulpitis) or, worse, a septic complication (periapical abscess or cellulitis) [45,46]. Taken together with the aforementioned findings, the high burden of advanced odontogenic infections and severe dental pathology within our cohort support the interpretation that the studied emergency dental unit frequently functions as a safety net, particularly for rural communities. However, this interpretation should be considered with nuance, as certain categories of emergencies—most notably maxillofacial trauma and temporomandibular joint injuries—may occur independently of routine dental attendance behaviors.

The peak of emergency presentation was observed in the evening, which aligns with previous findings [16,47,48,49]. This pattern may reflect the influence of socioeconomic factors, service accessibility, and behavioral timing considerations. Thus, engagement in work or school activities during daytime hours may delay care-seeking despite the onset of symptoms [50]. On the other hand, limited access to routine dental services outside standard working hours may contribute to increased reliance on emergency services during the evening period [51].

We also note that patients seeking emergency dental care in the morning/afternoon were significantly older. This may be related to a more rigorous scheduling or the severity of symptoms cumulated overnight [52]. Interestingly, the evening peak included not only the majority of patients but also most young individuals. This pattern may reflect scheduling constraints; more precisely, younger adults postpone dental visits during the day due to educational/professional activities and seek care after finishing their daily schedule. These findings suggest a potential association between younger age and later presentation times. This dynamic supports the need to adapt the medical personnel in this unit to accommodate increased patient flow during the second part of the day.

The temporal clustering of emergency presentations in weekends may stem from the limited availability of routine dental services during this time period. It may also reflect the progression of dental pain and infection over several days, leading to accumulation of untreated cases that subsequently present as emergencies. These findings are largely comparable with trends reported in previous studies [16,47,53]. Overall, our data indicate that emergency dental service utilization may be influenced not only by clinical severity but also by access to care, service availability, and patient behavior.

This study provides several important contributions to the existing literature. Our analysis of temporal dynamics across time intervals addresses a recognized gap in the international literature: information on when patients arrive (hours/day) is relatively underreported and insufficiently statistically validated in dental literature despite being essential for resource planning [19]. Studies that explicitly report time distributions frequently report peaks in the evening/early night—interpreted as an effect of outpatient service closures and schedule constraints of the working population [53]. The fact that our observation (peak in 16:00–24:00) aligns with these results strengthens the organizational interpretation, providing a local basis for staffing and triage recommendations.

In addition, the present results linking rural patients to increased odds for endodontic and periapical pathologies but decreased odds for other emergencies and unspecified causes constitute a differentiating signal compared to most Romanian studies that either do not find significant urban–rural differences in the distribution of diagnosis, or do not independently test this association (controlling for age/sex) [12,14,54]. The novelty is not that rural areas may have more difficult access; our observation suggests a change in composition of cases on the rural–urban axis towards more advanced pulpal and periapical diseases and less towards the heterogeneous non-specific dental emergencies, in an adjusted model. This type of signal is rarely explained in the available East European studies and is worth discussing as a hypothesis of mechanism (delay in presentation, differences in healthcare utilization patterns, distance, provider density).

Moreover, increasing age was associated with a significant decrease in the incidence of dental caries, but a corresponding increase in the incidence of complications. Petersen et al. show in the reports of World Health Organization that the incidence of dental caries remains elevated throughout lifetime, but its presentation form changes [55]. In this context, our data identify a critical window of intervention: at adult age, the simple dental caries become less frequent in emergency service presentations, being completely replaced by dental complications. These findings may indicate that, within this cohort, emergency dental services were more commonly utilized for advanced symptom management than for early-stage intervention.

Notwithstanding its important epidemiological strengths, this study is subject to several limitations that should be carefully considered. First, this was a retrospective, single-center study based on data derived from a single regional emergency dental department, which may limit generalizability to other geographic regions, institutional settings, or healthcare systems with differing demographic structures, referral pathways, and service accessibility. The observed diagnostic distributions and healthcare utilization patterns may therefore partially reflect local organizational or population-specific factors rather than broader national trends. Although this design offers valuable real-world insight into routine emergency dental practice, multicenter studies would be necessary to confirm external validity across diverse Romanian and international settings. Second, the relatively short study period of approximately three months may not fully capture seasonal or temporal fluctuations in emergency dental presentations, including variations associated with holidays, weather conditions, school schedules, occupational patterns, or temporary changes in outpatient service availability. Because this time frame may represent only a limited segment of annual healthcare demand, temporal bias is possible, and the observed attendance patterns may not fully reflect year-round epidemiology. As a result, extrapolation of these findings to broader annual trends should be approached with caution. Longer-term surveillance over multiple seasons or full-year periods would be particularly valuable to determine whether the identified patterns remain stable over time or are partially influenced by the specific study interval. Third, the pragmatic reliance on routinely recorded variables such as age, sex, and residence precluded inclusion of more comprehensive socioeconomic determinants—including education, income, employment, insurance status, prior dental attendance behavior, and systemic comorbidities—which may substantially influence healthcare access and emergency service utilization. Future Romanian studies should incorporate these dimensions to better distinguish structural healthcare barriers from behavioral presentation patterns. Fourth, the unit of analysis was the emergency presentation rather than the individual patient; while same-day duplicate presentations for identical diagnoses were excluded, separate visits on different days or for different conditions were intentionally retained to reflect real-world clinical workload, which may have modestly increased frequencies of certain recurrent diagnostic categories. Fifth, retrospective diagnostic coding based on existing clinical records introduces some potential for minor misclassification, particularly within broader heterogeneous categories such as “other emergencies,” which may combine diverse conditions with differing etiologies, prognoses, and long-term healthcare implications. Finally, exclusion of patients under 18 years of age limits assessment of pediatric and adolescent emergency dental patterns, potentially underrepresenting earlier stages of preventive care failure. Future multicenter, prospective, and longer-term studies incorporating broader socioeconomic, behavioral, and pediatric variables are therefore essential to strengthen generalizability, clarify structural healthcare barriers, and provide a more comprehensive understanding of temporal and population-level determinants of emergency dental healthcare utilization.

## 5. Conclusions

Our findings suggest that emergency dental services in Arad County serve an important role in managing predominantly advanced-stage dental pathology rather than early preventive presentations. Emergency care utilization was largely characterized by endodontic and periapical conditions, which comprised most cases. Increasing age was significantly associated with higher odds of more advanced pathology. Rural residence was associated with increased odds of endodontic and periapical pathology and decreased odds of other or unspecified conditions, while urban patients more frequently presented with non-specific emergencies. Emergency visits peaked during evening hours and weekends, particularly on Sundays and Saturdays. These results demonstrate substantial temporal and geographic variation in emergency dental presentations and may reflect disparities in disease progression and healthcare access. Improved alignment of staffing with peak demand periods and expanded preventive care accessibility, particularly in underserved rural areas, may help reduce emergency service dependence.

## Figures and Tables

**Table 1:** Classification of dental conditions and corresponding ICD-10 codes.

(Macro)Category	Description	Condition	ICD-10 Code(s)	*n* (%)
PD	Structural or caries-related, non-traumatic dental disease	Deep dental caries	K02.x	43 (8.43%)
EPP	Conditions primarily involving the dental pulp and periapical tissues	Acute apical periodontitis	K04.4	141 (27.65%)
		Chronic apical periodontitis	K04.5/K04.7	53 (10.39%)
		(including exacerbated)		
		Pulpitis	K04.0	103 (20.20%)
		(acute, serous, purulent, severe)		
OSC	Infectious conditions of odontogenic origin with local or systemic spread	Abscess	K12.2	56 (10.98%)
		(buccal, palatal, periodontal)		
		Cellulitis (genial, labial,	K12.2	53 (10.39%)
		submandibular)		
OE	Dental emergencies not included above or insufficiently classified	Osteoperiostitis/Osteitis	K10.2	24 (4.71%)
		Pericoronitis	K05.2/K05.3	12 (2.35%)
		(congestive, suppurative)		
		Root resorption (rhizolysis)	K03.3	8 (1.57%)
		Crown or root fracture	S02.5	6 (1.18%)
		(traumatic)		
		Hemorrhage	K06.8/T81.0	8 (1.57%)
		(post-implant, gingival bleeding)		
		Tooth eruption disorders	K00.6	2 (0.39%)
		Partially impacted tooth	K01.1	1 (0.20%)

PD, dental pathology; EPP, endodontic and periapical pathology; OSC, odontogenic infections and septic complications; OE, other emergencies.

**Table 2:** Sociodemographic parameters of dental emergency (macro)category.

(Macro)Category	Age	Sex (Male/Female)	Origin Area (Urban/Rural)
PD	20 (18; 27)	24 (55.81%)/19 (44.19%)	22 (51.16%)/21 (48.82%)
EPP	30 (22; 43)	156 (52.25%)/141 (47.25%)	160 (53.87%)/137 (46.23%)
OSC	29 (17; 42)	64 (58.71%)/45 (41.29%)	66 (60.55%)/43 (39.55%)
OE	31 (21; 42)	33 (54.09%)/28 (45.91%)	46 (75.40%)/15 (24.60%)

Data in the second column are given as median with upper and lower quartiles in parentheses. Data in third and fourth columns are presented as absolute counts with the corresponding percentages in parentheses. PD, dental pathology; EPP, endodontic and periapical pathology; OSC, odontogenic infections and septic complications; OE, other emergencies.

**Table 3:** Results of logistic regression for age, sex, and origin area across dental emergency macrocategories.

(Macro)Category	Predictor	β	SE	AOR (95% CI)	Wald (Z)	*p*
PD	Age	−0.062	0.013	0.94 (0.90; 0.98)	−4.61	<0.001 *
	Origin area (Rural)	0.385	0.341	1.47 (0.75; 2.91)	1.13	0.257
	Sex (Male)	0.344	0.347	1.41 (0.70; 2.83)	0.99	0.321
EPP	Age	0.049	0.017	1.05 (1.01; 1.12)	2.83	0.004 *
	Origin area (Rural)	0.399	0.186	1.49 (1.03; 2.15)	2.15	0.031 *
	Sex (Male)	−0.236	0.190	0.79 (0.55; 1.14)	−1.24	0.214
OSC	Age	−0.010	0.011	0.99 (0.98; 1.00)	−0.90	0.364
	Origin area (Rural)	−0.186	0.238	0.83 (0.54; 1.29)	−0.78	0.430
	Sex (Male)	0.199	0.212	1.22 (0.80; 1.89)	0.94	0.342
OE	Age	0.010	0.013	1.01 (0.99; 1.02)	0.77	0.440
	Origin area (Rural)	−0.942	0.320	0.39 (0.21; 0.73)	−2.94	0.003 *
	Sex (Male)	−0.051	0.283	0.95 (0.55; 1.63)	−0.18	0.857

β, beta coefficient; SE, standard error; AOR, adjusted odds ratio with 95% confidence interval; Wald(Z), z test value; PD, dental pathology; EPP, endodontic and periapical pathology; OSC, odontogenic infections and septic complications; OE, other emergencies. ‘Origin area (Rural)’ represents the effect of rural versus urban residence, while ‘Sex (Male)’ represents the effect of male versus female sex in the regression models. Bold values marked with asterisks (*) indicate significant predictors of specific dental pathologies (Wald test, ***—*p* ≤ 0.001; **—*p* ≤ 0.01; and *—*p* ≤ 0.05).

## Data Availability

The data presented in this study are available on request from the corresponding author due to privacy and ethical restrictions.

## Abbreviations

The following abbreviations are used in this manuscript:

PD	Dental pathology
EPP	Endodontic and periapical pathology
OSC	Odontogenic infections and septic complications
OE	Other emergencies and unspecified causes
AOR	Adjusted odds ratio
CI	Confidence interval (95%)
ICD-10	International classification of diseases, 10th revision
β	Beta coefficient (used in logistic regression)
SE	Standard error
Wald (Z)	Wald statistic/Z-test value
EU	European Union
WHO	World Health Organization
